# A Calorie-Restricted DASH Diet Reduces Body Fat and Maintains Muscle Strength in Obese Older Adults

**DOI:** 10.3390/nu12010102

**Published:** 2019-12-30

**Authors:** Cydne A. Perry, Gary P. Van Guilder, Alyssa Kauffman, Mosharraf Hossain

**Affiliations:** Department of Health and Nutritional Sciences, South Dakota State University, Brookings, SD 57007, USA; gary.vanguilder@sdstate.edu (G.P.V.G.); alyssa.kauffman@sdstate.edu (A.K.); mosharraf.hossain@sdstate.edu (M.H.)

**Keywords:** older adults, DASH diet, calorie restriction, protein intake, body composition, muscle

## Abstract

This study examined the effect of the Dietary Approaches to Stop Hypertension (DASH) diet containing lean red meat on measures of body composition and muscle strength in a cohort of obese adults 65 and older; 36 males (*n* = 15) and females (*n* = 21) consumed 1800 kcal/day for 12 weeks under controlled feeding conditions. The study diet included daily intakes of 126 g of meat. Measures of body composition and muscle strength were obtained at weeks 0, 3, 6, 9, and 12. Breakfast, lunch, and dinner were provided every day for 12 weeks, and equal portions of meat were distributed at each meal. Significant effects of the study diet were detected across time for total body weight, body mass index (BMI), waist circumference, hip circumference, body fat percentage, absolute fat mass (AFM), and blood pressure such that a decrease (*p* < 0.001) was observed over 12 weeks. Significant effects of the study diet were detected across time for sit/stand (*p* < 0.001) such that an increase was observed. From baseline to study end, total body weight decreased by 6.3% (*p* < 0.001), body fat percentage decreased by 2.5% (*p* < 0.001), and absolute fat mass (AFM) decreased by 4.4 kg (*p* < 0.001). By the study end, skeletal muscle mass (SMM) was positively correlated with handgrip strength (R^2^ = 0.75; *p* = 0.001) and resting energy expenditure (REE) (R^2^ = 0.29; *p* = 0.001). Handgrip strength, gait, balance, and resting energy expenditure (REE) were well maintained (*p* > 0.05) throughout the study. These findings suggest that the DASH diet has the potential to be a tool to preserve muscle strength while reducing fat mass in obese older adults.

## 1. Introduction

Age-related declines in muscular mass resulting in sarcopenia is an important determinant of physical function, strength, and performance in older adults. As a result, decreased walking ability, increased fall risk, and disability (e.g., hip/leg fractures and head trauma) occur, leading to increased hospitalizations and rates of in-home care, overcrowding of assisted living facilities, declines in independence and quality of life, burdens on family members, and increased health-care costs. Within this population, an accumulation of fat mass occurs simultaneously with reduced muscle mass and strength leading to metabolic dysregulation, resulting in accelerated disease onset and increased mortality. Maintaining muscle mass and strength while reducing fat mass accumulation are vital to maintaining mobility and reducing disease risk.

Diet quality and dietary protein intake are vital for maintaining body composition and muscle mass, as well as improving physical performance [1]. Malnutrition in dietary protein intake is a major cause of reduced muscle mass, strength, and function in older adults [2]. The current recommended dietary allowance (RDA) for protein for adults is 0.8 g/kg/day, which averages to ~50 g/d (56 g/d for men; 46 g/d for women) for average weight sedentary adults [3]. Consuming less than the protein RDA results in significant declines in muscle mass, strength, and function in older populations [4,5], and to preserve muscle mass and strength, it is recommended that older adults consume at least 1.1–1.3 g/kg of protein daily [4,5,6,7]. Older adults who maintain a high-quality diet with adequate protein intake have reduced health abnormalities related to muscle and fat mass [6]. The Dietary Approaches to Stop Hypertension (DASH) dietary pattern is a high-quality therapeutic diet known to improve health status in various diverse and at-risk populations resulting in improved heart health, maintained cognitive function, and reductions in metabolic diseases such as diabetes, metabolic syndrome, and nonalcoholic fatty liver disease [8,9,10,11,12]. The primary protein recommendations of the DASH diet are poultry and fish, and it is recommended to decrease red meats from the diet. However, studies have demonstrated that lean red meat incorporated into a DASH-like diet does not exacerbate cardiovascular health indices in adults [13,14], indicating that lean red meat can be included in the DASH diet without negative effects on heart health. Although studies have reported on the DASH diet in older adults, no studies have examined the influence of the DASH diet containing lean red meat on measures of body composition and muscle mass under controlled feeding conditions. The purpose of this study was to evaluate the effect of the DASH diet that includes daily intakes of lean red meat on a comprehensive assessment of body composition and muscular fitness in adults 65 and older.

## 2. Materials and Methods

### 2.1. Study Participants

Adults aged 65 years and older were recruited from the rural community of Brookings, SD between June 2017 and August 2018 by the use of approved flyers posted in the local newspaper and at South Dakota State University. During the screening phase, all study volunteers completed a questionnaire that included the date of birth, medication use, vitamin and mineral use, and drug and alcohol use. Entry into this study was contingent upon age and upward mobile ability. Additional inclusion criteria included (i) eating one meal per day at the on-site location, (ii) not consuming foods and beverages outside those provided by research personnel, and (iii) undergo body composition and muscle fitness measurements. The study was conducted in accordance with the Declaration of Helsinki. The protocol was reviewed and approved by the Institutional Review Board for Human Study Participant Use at South Dakota State University (Approval number: IRB-1712006-EXP) and informed consent was obtained from all participants before entry into the study (ClinicalTrials.gov Identifier: NCT04127240).

### 2.2. Study Design and Diet

The study was a 12-week controlled-feeding dietary intervention in which males (*n* = 15) and females (*n* = 21) aged 65 years and older consumed 126 g of fresh, lean red meat (beef or pork) per day as a part of the DASH dietary pattern. This diet was created using Nutritionist Pro software and based upon the 2015–2020 dietary guidelines for daily caloric intake for older sedentary adults [15] and the DASH eating plan by the National Heart, Lung, and Blood Institute, National Institutes of Health [16] (Table 1). The study diet provided equal amounts of meat at the three major meals: breakfast (42 g), lunch (42 g), and dinner (42 g). All food items were purchased by research staff from the local grocery store (Hy-Vee, Inc., Brookings, SD, USA) and were weighed out to the nearest gram and prepared in the food’s laboratory at South Dakota State University. Study participants were required to consume at least one meal per day in the food’s laboratory Monday through Friday; all other meals, snacks, and beverages were provided as takeaways. The caloric intake for this study was 1800 kcal/day, which was based upon the 2015–2020 dietary guidelines for daily caloric intake for sedentary adults aged 61 years and older [15]. The guidelines recommend 2000 kcal/day for men and 1600 kcal/day for women. For the purposes of this study and the controlled-feeding design, the average of the caloric intakes for men and women within this age group was used. The DASH eating plan for an 1800 kcal diet was used to create the composition of the study diet [16] (Table 1). The investigators had daily contact with participants throughout the study, which enhanced the compliance of participants to the dietary regimen. In addition, participants were required to verify the consumption of each food item by completing a daily checklist provided by the investigators. A multivitamin/multimineral supplement for seniors (Hy-Vee Health Market Senior Multivitamin & Multimineral Supplement) was provided daily to ensure adequate micronutrient intake. Overall, the study protocol was well-tolerated with the study regimen completed by 80% of participants. Reasons for stopping the study included surgery (*n* = 1), illness (*n* = 2), personal challenges (*n* = 4), and challenges adhering to study protocol (*n* = 1).

### 2.3. Follow-Up Visits

All measures (described below) were collected at five time points throughout the feeding study: weeks (wks) 0 (baseline), 3, 6, 9, and 12. Follow-up visits occurred in the morning following an overnight fast.

### 2.4. Hemodynamic Measurements

Auscultatory resting systolic and diastolic blood pressure were measured using a stethoscope and sphygmomanometer (Diagnostic 700 Series; American Diagnostic Corporation, Hauppauge, NY, USA) following 5 min of seated quiet rest using standard procedures. Resting blood pressure measurements were performed twice separated by 3 min and averaged. Resting heart rate was measured using a 60 s radial pulse count.

### 2.5. Body Composition

Height was measured using a medical beam balance (Seca Corporation, Hamburg, Germany). Body mass index was calculated as weight (kilograms) divided by height (meters) squared. Abdominal waist circumference was assessed with a Gulick tape measured at the smallest part of the abdomen, above the umbilicus, and below the xiphoid process to the nearest 0.1 cm at the end of normal expiration using standard procedures. Hip circumference was measured similarly at the maximal circumference of buttocks. Waist and hip measurements were measured in duplicate and averaged. Weight, percent body fat, absolute fat mass (AFM), fat free mass (FFM), skeletal muscle mass (SMM), and total body water (TBW) were measured by bioelectrical impedance (InBody 270, InBody, Cerritos, CA, USA).

### 2.6. Muscle Fitness and Function

All muscle fitness and function assessments were conducted using strict adherence to the American and British Geriatric Societies’ Clinical Practice Guidelines [17] and the Centers for Disease Control and Prevention STEADI (Stopping Elderly Accidents, Deaths, and Injuries) Tool Kit [18] for the prevention of falls in older persons. For each assessment, participants were provided instructions regarding the proper procedure and outcome. This was followed by a series of demonstrations provided by the research team. Thereafter, participants performed the assessment to practice and familiarize themselves to the procedure. Corrections, adjustments to form, and additional instructions were then provided to ensure proper completion of the measurement.

Handgrip strength (kg) was quantified by the maximum grip force of the right and left hand using a hand-held dynamometer (Smedley III analog). Right and left grip strength data were summed to provide a composite score. Grip strength was classified from normative scores established by the American College of Sports Medicine’s guidelines for exercise testing [19]. Grip strength relative to body weight was calculated by dividing grip force by the body mass (kg) of the participant at each time point.

Resting energy expenditure (REE) was estimated using open-circuit spirometry combined with indirect calorimetry (Parvo Medics TrueOne^®^ 2400, Salt Lake City, UT, USA) following an overnight fast with no caffeine in a quiet temperature and humidity-controlled exercise physiology laboratory. Flow and gas calibration were performed prior to each test using standard operating procedures provided by the manufacturer. Participants were equipped with a mouthpiece, nose clip, and a heart rate monitor affixed to the chest with receiver integrated with the metabolic cart (Polar Electro Inc., Lake Success, NY, USA). Baseline expired gases were collected for 5 min to establish steady-state conditions. Thereafter, oxygen consumption and calories expended were measured for 15 min while seated quietly at rest. Oxygen uptake data were smoothed with a 15-breath moving average. The 15-breath moving average was selected because it induces minimal data loss with little data and trend distortion, as recommended by Robergs et al. [20]. REE data during the final 2 min of the 20 min rest period were used for analysis. REE data was expressed as the total energy expenditure per day, relative to body weight (kg), and relative to absolute fat mass (AFM).

The 30 s sit-to-stand test, a component of the Senior Fitness Test Battery and the Fullerton Functional Fitness Battery [21], was used to determine lower extremity strength and functional balance and mobility (e.g., lower body mobility needed to climb stairs, get out of a car, chair, and tub etc.) in older adults (≥65 years).

### 2.7. Gait and Balance Analysis

A series of walking and stability measurements were conducted using the Tinetti Performance-Oriented Assessment of Mobility Problems in Elderly Patients [22] and the Fullerton Advanced Balance Scale [23]. The Tinetti assessment consisted of nine balance indicators and seven gait indicators that were scored separately on a three-point ordinal scale (0 to 2). These scores were summed to provide an overall balance/gait score. A score of <19 out of 28 indicates increased fall risk. The Fullerton balance scale consisted of 10 performance-based activities that were scored on a five-point ordinal scale (0–5) with higher scores indicating better gait and balance ability. A score of 25 or lower indicates a high risk of falls [24]. Both tests are sensitive indicators of balance and gait decrements in older persons and are sensitive to clinical improvements in response to physical therapy interventions [25].

### 2.8. Statistical Analysis

Data were checked for normality and spread with the Shapiro–Wilk test and were considered normally distributed. Differences in baseline characteristics between males and females were determined by one-way ANOVA. A general linear model one-way repeated measures ANOVA was use for within-group comparisons in the dependent variables resulting from the dietary intervention. The within-subjects factor was time (wks 0, 3, 6, 9, and 12) with the primary outcome of interest being the difference between baseline and week 12. When indicated by a significant *F* value, pairwise differences at specific time points were identified using the Bonferroni adjustment for multiple comparisons. To adjust for the influence of changes in body weight across the intervention, we repeated the analyses of the time effect for muscle strength and function outcomes by including the percent change in body weight (from baseline to study end) as a covariate in general linear model repeated measures ANOVA. In addition to pooling data for males and females, data are displayed separately by sex. Relations between SMM and indicators of muscle strength and function at study end were assessed by Pearson’s correlation. Statistical significance was set at *p* < 0.05. Data are presented as means (SD) and analyzed with SPSS version 24 (IBM Inc., Armonk, NY, USA).

## 3. Results

### 3.1. Participant Characteristics and Baseline (wk 0) Measurements

Thirty-six participants aged 70 years (range = 65–84 years) completed the 12-week controlled-feeding study and were included in the final analysis. Baseline differences (*p* < 0.05) between males (*n* = 15) and females (*n* = 21) were detected for height, weight, waist, WHR, body fat (%), FFM, LBM, SMM, TBW, REE, and handgrip. No differences (*p* > 0.05) were detected for age, body mass index (BMI), hip, systolic blood pressure, sit/stand, gait and balance (Table 2).

### 3.2. Effects of the DASH Diet on Indicators of Body Composition

Throughout the 12-week study period, changes in body composition were detected with significant effects of the diet across time detected for body weight (*p* < 0.001); BMI (*p* < 0.001); waist (*p* < 0.001); hip (*p* < 0.001); body fat percentage (*p* < 0.001); AFM (*p* < 0.001); FFM (*p* < 0.001); LBM (*p* < 0.001); and TBW (*p* < 0.001) such that a decrease was observed in all participants over the 12 weeks (Table 3).

In all participants, body weight decreased (*p* < 0.001) by 6.3% from baseline (91.2 kg) to study end (85.5 kg); BMI decreased (*p* < 0.001) from baseline (32.0) to study end (30.1); waist decreased (*p* < 0.001) from baseline (101 cm) to study end (96.3 cm); hip decreased (*p* < 0.001) from baseline (115 cm) to study end (111 cm); body fat percentage decreased (*p* < 0.001) from baseline (37.2%) to study end (34.7%); AFM decreased (*p* < 0.001) from baseline (34.5 kg) to study end (30.3 kg); and LBM decreased (*p* < 0.05) from baseline (64 kg) to study end (62 kg). In males only, WHR decreased (*p* < 0.05) from baseline (0.97) to study end (0.95) (Table 3). At baseline, 32 participants entered the study as obese (*n* = 24) or overweight (*n* = 8) based upon BMI category. By study end, 31% of the participants reduced enough body mass, resulting in an overweight (*n* = 7) or normal weight (*n* = 3) BMI category.

### 3.3. Effects of the DASH Diet on Measurements of Muscle Fitness

Throughout the 12-week study period, indicators of muscle and metabolic health were influenced by the study diet with significant effects of the diet across time. A decrease was observed in all participants for SMM (*p* < 0.001), systolic blood pressure (*p* < 0.001), and diastolic blood pressure (*p* < 0.001). Over the course of the study, there was an increase (*p* < 0.001) in the sit/stand measurement, and in females only, handgrip strength increased (*p* = 0.058) over the 12-week period. In all participants, handgrip, gait, balance, and REE were well maintained (*p* > 0.05).

**Figure 1:**
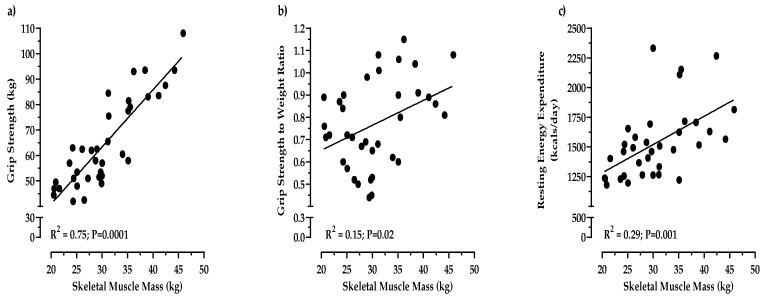
Bivariate correlations at study-end between skeletal muscle mass and handgrip strength (**a**), handgrip strength to body weight ratio (**b**), and resting energy expenditure (**c**).

In all participants, the magnitude of fat loss (4.4 kg) at the end of 12 weeks was fivefold greater (*p* < 0.0001) compared to the loss of muscle mass (0.8 kg). While we observed a 0.8 kg decrease in SMM from 31.4 kg at baseline to 30.6 kg at study end, muscle strength was well maintained. In fact, from baseline to study end, handgrip strength increased (*p* < 0.0001), even though participants lost over 6% of their total body weight. As a result, we also observed that REE was well maintained, and increased (*p* < 0.0001) as absolute fat mass decreased (Table 4). Moreover, muscle function as demonstrated by the 30 s sit/stand test significantly improved (*p* < 0.001) from baseline (11.4) to study end (13.8) (Table 4). The favorable changes in handgrip muscle strength, REE, and the 30 s sit/stand test remained after controlling for the decrease in body weight. By the study end, SMM was positively associated with handgrip strength (R^2^ = 0.75, *p* = 0.001) and REE (R^2^ = 0.29, *p* = 0.001) (Figure 1). There were also significant decreases in systolic blood pressure (*p* < 0.001) from baseline (133 mmHg) to study-end (120 mmHg); meanwhile, diastolic blood pressure decreased (*p* < 0.005) from baseline (76.3 mmHg) to study end (70.5 mmHg). The decreases in blood pressure remained significant after adjusting for changes in total body weight.

## 4. Discussion

This highly controlled feeding study sought to examine the effects of a calorie-restricted DASH diet containing daily intakes of fresh lean red meat on changes in body composition and muscular strength in adults 65 and older. The following two main findings emerged:

(1) Daily consumption of the study diet altered a comprehensive panel of body composition measurements in obese older adults.
(2) Daily meat as a part of the DASH diet was associated with preserved handgrip strength, a significantly increased strength-to-weight ratio, and increased resting energy expenditure in adults 65 and older.

### 4.1. Body Composition Improved in Obese Older Adults Consuming the DASH Diet

In the present study under controlled dietary intakes, total body weight was significantly reduced by 6.3% (*p* < 0.001) at a rate of 0.5 kg (1.1 lb) per week as a result of consumption of the study diet over the course of 12 weeks. The loss of total body weight greatly contributed to the reductions in other measures of body composition including BMI, body fat percentage, and AFM (*p* < 0.001). The 0.5 kg per week of weight loss follows the Centers for Disease Control and Prevention (CDC) recommendation of gradual and steady weight loss of 1–2 lbs per week for long-term success [26]. Although the average BMI for all participants by study end was 30.1 kg/m^2^ (obese class I category), the 6.3% reduction in total body weight suggests that the risks associated with chronic disease onset is reduced [27]. Notably, 31% of the participants reduced enough body mass to shift to a less severe BMI category by study end, and the outcomes of this study showed similar trends in all measurements for both males and females.

Of the factors related to total body weight, the greatest contributing factor to the reduction in total body weight was the loss of body fat. In the present study, all participants on average lost 4.4 kg of fat mass (*p* < 0.001) resulting in 27.6% (*p* < 0.001) body fat for males and 39.8% (*p* < 0.001) body fat for females by study end. This loss of body fat would imply an improved health status; however, given that there are no established cutoff levels for body fat that define obesity in males and females, it is impossible to associate these results to health outcomes. A recent review by JafariNasabian [28] suggested a 32%–35% body fat cutoff for obesity for women, which is lower than the proposed 40% cutoff by the World Health Organization [29]. However, Liu et al. suggests that women maintain body fat at 30% or lower to preserve bone health [30]. Certainly, more studies are required to determine body fat levels that define obese phenotypes in males and females and determine the relationships of such levels with poor health outcomes.

The decrease in body fat observed in the present study is related to the 5 cm reduction in waist circumference (*p* < 0.001), which serves as a surrogate indicator of obesity-related chronic disease risk [31,32]. The accumulation of abdominal body fat is the hallmark of the obese phenotype in older adults. Abdominal fat accumulation is the primary contributing factor to the development of obesity-related chronic diseases afflicting this population and promotes the onset of sarcopenia and osteopenia [33]. According to the CDC, 41% of older adults in the United States are obese, and the National Council on Aging states that 80% of older adults have at least one chronic disease [34,35]. The economic burden of chronic diseases within the U.S. is approximately $170 billion per year, which accounts for 17% of total health care costs [36]. Given the outcomes of this study within this small cohort of older adults, the DASH diet may be a potential method to improve body composition that may in turn lead to obesity management and reduce disease risk in obese older adults.

In the present study, systolic and diastolic blood pressure were drastically reduced. At baseline, blood pressure was on average 133/76 mmHg for all participants, which is a category of high blood pressure (hypertension) Stage 1. Importantly, by study end, the average blood pressure was in the healthy range of 120/70 mmHg and many of the participants eliminated high blood pressure as a risk factor. The significant reduction in blood pressure remained even after controlling for weight loss. Nevertheless, we cannot fully discount the likely benefit of body weight loss (and significantly reduced central adiposity) on the decreases in blood pressure in the present study. However, the composition of the study diet, which was based upon the DASH dietary pattern (i.e., an emphasis on the consumption of foods that are low in sodium and high in magnesium, potassium, and calcium) may also have contributed to the favorable modifications in blood pressure. Indeed, controlling for weight loss did not alter the beneficial drop in blood pressure. The study diet consumed by all participants contained foods that provided daily average dietary intakes of 1895 mg of sodium, 585 mg of magnesium, 4395 mg of potassium, and 1187 mg of calcium. Additionally, all participants consumed a daily multivitamin/multimineral supplement that provided an additional 50 mg of magnesium, 80 mg of potassium, and 220 mg of calcium. Collectively, despite weight loss, our findings suggest that the diet provided the required mineral composition to support the reduction in blood pressure observed within this population and supports the effectiveness of the DASH diet to lower blood pressure in obese adults with hypertension.

### 4.2. Muscle Strength was Maintained in Obese Older Adults Consuming Meat as a Part of the DASH Diet

Very recently, the European Working Group on Sarcopenia in Older People (EWGSOP) has redefined sarcopenia to be a disease of muscle failure with muscle strength as the primary determinant of sarcopenia [37]. According to the EWGSOP, muscle strength is now considered the most reliable measure of muscle function in older adults, superseding the role of muscle mass. Additionally, grip strength is a dominant predictor of poor health outcomes in older adults such as poor quality of life, increased hospitalizations, reduced functional capabilities, and death [38,39]. In the present study, despite the loss in total body mass, grip strength was well preserved (*p* > 0.05) in all participants over the course of 12 weeks with an increase observed in females (*p* = 0.058). Furthermore, in all participants, we observed an increase in relative body strength. That is, as body mass decreased over time, grip strength increased (<0.0001) during the study period, indicating that participants’ strength-to-weight ratio was significantly improved. This is critically important, because reduced walking mobility and activities of daily living in obese older adults is due in part to poor strength-to-weight ratio, which may increase the risk of falls and subsequent mobility disability. By improving the strength to weight ratio, an older obese person will be able to perform activities of daily living, including walking and stair climbing at a lower percentage of their strength capacity. Thus, the physical effort needed to perform everyday activities is less. In addition, although grip strength alone serves as a dominant predictor of poor health outcomes in older adults, gait and sit/stand serve as additional indicators of physical performance and strength. Gait is a measure of physical performance that has been shown to be a predictor for adverse outcomes related to sarcopenia such as falls, cognitive impairment, disability, and mortality [40,41,42,43]. Moreover, sitting and standing requires strength and endurance and serves as a proxy for leg muscle strength. In the present study, gait was well preserved (*p* > 0.05) and sit/stand increased (*p* < 0.001) over the course of 12 weeks. Furthermore, related to gait and sit/stand, balance was also well maintained (*p* > 0.05). The present study also demonstrated that total body energy expenditure was well maintained (*p* > 0.05) throughout the study, and by the study end, the total body energy expenditure was moderately associated with skeletal muscle mass (R^2^ = 0.29, *p* = 0.001). In fact, per kg of body fat and muscle mass, energy expenditure was increased, even though the total amount of muscle mass and body weight were reduced. Such a finding is clinically important, as it suggests that the DASH diet coupled with high-quality protein from lean red meat increases the efficiency to maintain muscle strength and energy expenditure. Taken together, these findings show that despite the loss of total body mass, measures of muscular strength, muscle function, and resting energy expenditure were maintained or increased as a result of consuming daily intakes of 126 g of high-quality meat as a part of the study diet, suggesting an improved health status over time and support the updated definition of sarcopenia by the EWGSOP.

In addition to dietary protein intake, micronutrients provided by the study diet and the supplement, such as vitamin D, vitamin B_12_, and iron may have also played a role in the outcomes related to muscle strength. The study diet provided 4.7 mcg of vitamin D, 7.3 mcg of vitamin B_12_, and 14 mg of iron. The multivitamin/multimineral supplement provided an additional 12.5 mcg of vitamin D and 25 mcg of vitamin B_12_.

### 4.3. Limitations

A major limitation of this study is the lack of a comparison group; thus, the outcomes of this study cannot be evaluated against a different dietary pattern. Moreover, as all of the participants on this study were sedentary and obese, physical activities beyond activities of daily living were not monitored. This was a small cohort of older adults that were all Caucasian and reside in the rural community of Brookings, SD. Furthermore, all of the participants lived in their own homes, were upwardly mobile, and none of the participants required assistance for daily living activities nor lived in assisted living facilities. Due to these limitations, additional studies are required, and caution should be used when generalizing the outcomes of the present study to populations of older adults with differing ethnic/racial and demographic backgrounds as well as various living conditions.

## 5. Conclusions

In conclusion, the outcomes of this study suggest that the DASH dietary pattern has the potential to be an effective tool to reduce body fat and manage obese phenotypes in adults 65 and older. The present findings suggest that daily intakes of high-quality protein consumed throughout the day aids in the maintenance of strength, performance, and total body energy expenditure. The present study supports the recommendation that older adults should consume protein above the current RDA, and suggests the consumption of at least 126 g of high-quality protein per day as a part of a healthy dietary pattern to maintain muscle strength and performance. The findings of this study also support the 2015–2020 dietary guidelines for caloric intake for older sedentary adults, particularly those with obese phenotypes.

## Figures and Tables

**Table 1:** Study diet compared to Dietary Approaches to Stop Hypertension (DASH) recommended servings.

Food Group	DASH Recommended Servings	Study Diet
Grains	6	7
Vegetables	4–5	5
Fruits	4–5	4
Dairy	2–3	3
Lean Meat	6 or less	4.5
Legumes	4 per week	4 per week
Fats/Oil	2–3	3
Sweets	5 or less	0
Sodium	2300 mg/day	1895 mg/day
Calories	1800	1800 cal/day
Macronutrients	Carbohydrates: 45%–65%	59%
	Fat: 20%–30%	21%
	Protein: 15%–25%	20%
Saturated Fat	<6%–10%	8%

Note: The study diet was created using the DASH eating plan and the 2015–2020 dietary guidelines for estimated caloric needs. www.nhlbi.nih.gov/health-topics/dash-eating-plan. https://health.gov/dietaryguidelines/2015/guidelines/appendix-2/.

**Table 2:** Baseline characteristics of study participants. AFM: absolute fat mass, BMI: body mass index, DBP: diastolic blood pressure, FFM: fat free mass, LBM: lean body mass, REE: resting energy expenditure, SBP: systolic blood pressure, SMM: skeletal muscle mass, TBW: total body water, WHR: waist-to-hip ratio.

Variables	Total (*n* = 36)	Male (*n* = 15)	Female (*n* = 21)	*p* Value
Age (year)	70.7	71.5	70.1	0.473
Height (cm)	169.1	177.3	163.2	**<0.0001**
Weight (kg)	91.2	98.7	85.9	**0.033**
BMI (kg/m^2^)	32.0	31.4	32.5	0.646
Waist (cm)	100.7	108.1	95.4	**0.020**
Hip (cm)	115.3	111.0	118.3	0.125
WHR	0.874	0.974	0.803	**<0.0001**
Body Fat (%)	37.2	30.7	41.8	**<0.0001**
AFM (kg)	34.5	30.5	37.3	0.116
FFM (kg)	56.7	68.1	48.6	**<0.0001**
LBM (kg)	64.0	71.1	58.9	**<0.0001**
SMM (kg)	31.4	38.0	26.6	**<0.0001**
TBW (kg)	40.7	49.7	34.2	**<0.0001**
REE (kcal/day)	1600.3	1785.9	1442.1	**0.002**
REE (kcal/day/kg)	17.8	18.2	17.5	0.504
SBP (mmHg)	132.9	131.2	134.1	0.671
DBP (mmHg)	76.3	79.9	73.7	0.056
Handgrip (kg)	62.6	78.9	51.1	**<0.0001**
Sit/Stand (reps)	11.4	11.9	11.1	0.364
Gait score	11.6	11.8	11.5	0.167
Balance score	14.4	14.1	14.7	0.365

Note: Data are expressed as means (sd) for one-way ANOVA. The bold distinguishes significance from non-significance at baseline.

**Table 3:** Body composition changes in older adults consuming the DASH diet for 12 weeks.

	Weeks of Intervention					
Variable	0	3	6	9	12	*p*-Value ^+^
Weight (kg)						
All Participants	* 91.2 (18.0)	88.6 (17.3)	87.3 (16.9)	86.4 (16.6)	* 85.5 (16.3)	<0.001
Females	* 85.9 (19.9)	84.0 (19.3)	82.9 (18.9)	82.0 (18.4)	* 81.3 (18.2)	<0.001
Males	* 98.7 (11.8)	95.0 (11.9)	93.4 (11.6)	92.5 (11.5)	* 91.5 (11.1)	<0.001
BMI (kg/m^2^)						
All Participants	* 32.0 (6.9)	31.2 (6.7)	30.7 (6.6)	30.4 (6.5)	* 30.1 (6.4)	<0.001
Females	* 32.5 (8.5)	31.8 (8.3)	31.4 (8.2)	31.0 (8.0)	* 30.8 (7.9)	<0.001
Males	* 31.4 (3.6)	30.3 (3.6)	29.7 (3.5)	29.4 (3.5)	* 29.1 (3.3)	<0.001
Waist (cm)						
All Participants	* 101 (16.4)	99.1 (15.7)	97.9 (15.9)	97.0 (15.5)	* 96.3 (15.1)	<0.001
Females	* 95.4 (18.4)	94.3 (17.8)	93.2 (18.0)	92.3 (17.7)	* 92.1 (17.4)	<0.001
Males	* 108.1 (9.2)	105.8 (8.7)	104.5 (9.2)	103.7 (8.7)	* 102.2 (8.6)	<0.001
Hip (cm)						
All Participants	* 115 (14.0)	114 (14.1)	113 (14.3)	112 (13.2)	* 111 (14.1)	<0.001
Females	* 118 (16.7)	117 (16.6)	116 (17.0)	114 (15.6)	* 114 (16.6)	<0.001
Males	* 111.0 (7.9)	109.7 (8.2)	109.0 (8.2)	108.9 (8.2)	* 107.3 (8.3)	<0.05
WHR						
All Participants	0.87 (0.10)	0.87 (0.10)	0.87 (0.10)	0.86 (0.09)	0.86 (0.09)	0.06
Females	0.80 (0.06)	0.80 (0.06)	0.80 (0.06)	0.80 (0.06)	0.80 (0.06)	>0.05
Males	* 0.97 (0.05)	0.96 (0.05)	0.96 (0.05)	0.95 (.05)	* 0.95 (0.05)	<0.05
Body Fat (%)						
All Participants	* 37.2 (9.8)	36.9 (10.1)	35.7 (10.1)	35.3 (10.4)	* 34.7 (10.3)	<0.001
Females	* 41.8 (9.5)	41.7 (9.7)	40.4 (9.8)	40.3 (9.9)	* 39.8 (9.6)	<0.001
Males	* 30.7 (5.8)	30.2 (6.1)	29.2 (6.2)	28.2 (6.5)	* 27.6 (6.4)	<0.001
AFM (kg)						
All Participants	* 34.5 (12.7)	33.4 (12.9)	31.9 (12.6)	31.1 (12.7)	* 30.3 (12.4)	<0.001
Females	* 37.3 (14.8)	36.4 (14.8)	34.9 (14.6)	34.4 (14.4)	* 33.7 (14.1)	<0.001
Males	* 30.5 (7.9)	29.1 (8.3)	27.5 (7.8)	26.4 (8.0)	* 25.5 (7.7)	<0.001
FFM (kg)						
All Participants	56.7 (12.2)	55.3 (11.3)	55.4 (11.2)	55.3 (11.4)	55.2 (11.3)	<0.05
Females	48.6 (7.0)	47.6 (6.3)	48.0 (6.1)	47.6 (5.9)	47.5 (5.8)	<0.05
Males	68.1 (8.0)	65.9 (7.1)	65.9 (7.7)	66.1 (7.9)	66.0 (7.8)	<0.05
LBM (kg)						
All Participants	* 64.0 (10.5)	62.3 (9.6)	62.4 (9.5)	62.2 (9.7)	* 62.0 (9.6)	<0.001
Females	* 58.9 (9.0)	57.7 (8.2)	57.9 (8.0)	57.4 (7.8)	* 57.3 (7.6)	<0.001
Males	71.1 (8.2)	68.8 (7.4)	68.7 (8.0)	68.9 (8.2)	68.7 (8.0)	<0.001
TBW (kg)						
All Participants	41.5 (8.8)	40.5 (8.3)	40.7 (8.2)	40.6 (8.4)	40.5 (8.3)	<0.001
Females	35.7 (5.2)	34.9 (4.6)	35.2 (4.5)	34.9 (4.4)	34.9 (4.3)	0.007
Males	49.7 (5.9)	48.4 (5.2)	48.3 (5.6)	48.5 (5.8)	48.3 (5.7)	0.003

Note: Data are expressed as mean (SD) for general linear model repeated-measures ANOVA with Bonferroni adjustment for multiple comparisons. + *p* value for the effect of diet across time. * *p* < 0.05 for the difference between baseline and study end.

**Table 4:** Muscle and metabolic health in older adults consuming the DASH diet containing meat.

	Weeks of Intervention					
Variable	0	3	6	9	12	^+^*p*-Value
Skeletal muscle (kg)						
All Participants	* 31.4 (7.2)	30.7 (6.7)	30.8 (6.6)	30.7 (6.8)	* 30.6 (6.8)	<0.001
Females	26.6 (4.1)	26.1 (3.6)	26.4 (3.5)	26.0 (3.4)	26.0 (3.4)	<0.05
Males	38.0 (4.9)	37.1 (4.3)	37.0 (4.6)	37.2 (4.8)	37.0 (4.8)	<0.05
Handgrip (kg)						
All Participants	62.6 (19.4)	62.8 (17.1)	64.1 (18.0)	62.9 (17.3)	64.7 (17.5)	>0.05
Females	51.1 (9.7)	51.8 (7.7)	52.5 (8.6)	51.6 (9.0)	53.0 (7.1)	0.058
Males	78.8 (18.0)	78.3 (14.6)	80.4 (14.8)	78.8 (13.1)	81.0 (14.2)	>0.05
Handgrip (per kg mass)						
All Participants	* 0.70 (0.21)	0.73 (0.20)	0.75 (0.20)	0.74 (0.19)	* 0.77 (0.19)	<0.0001
Females	* 0.62 (0.18)	0.65 (0.17)	0.66 (0.17)	0.66 (0.17	* 0.68 (0.16)	<0.0001
Males	* 0.81 (0.21)	0.84 (0.19)	0.87 (0.19)	0.86 (0.17)	* 0.90 (0.17)	<0.0001
Sit/Stand (reps)						
All Participants	* 11.4 (2.3)	11.9 (2.4)	12.9 (2.5)	13.1 (2.9)	* 13.8 (2.5)	<0.001
Females	* 11.1 (2.1)	11.6 (2.3)	12.5 (2.3)	12.8 (2.8)	* 13.4 (2.1)	<0.001
Males	* 11.9 (2.6)	12.4 (2.4)	13.4 (2.8)	13.5 (3.0)	* 14.3 (3.0)	<0.001
Gait score						
All Participants	11.6 (0.7)	11.6 (0.7)	11.8 (0.5)	11.9 (0.4)	11.8 (0.5)	>0.05
Females	11.5 (0.8)	11.5 (0.9)	11.8 (0.5)	11.9 (0.3)	11.9 (0.5)	>0.05
Males	11.8 (0.4)	11.8 (0.4)	11.9 (0.4)	11.8 (0.6)	11.8 (0.6)	>0.05
Balance score						
All Participants	14.4 (1.7)	15.0 (1.3)	15.3 (0.8)	15.1 (1.1)	15.1 (1.1)	>0.05
Females	14.7 (1.4)	15.2 (0.9)	15.3 (0.9)	15.1 (1.1)	15.0 (1.2)	>0.05
Males	14.1 (2.1)	14.7 (1.6)	15.3 (0.8)	15.1 (1.1)	15.3 (1.0)	>0.05
REE (kcal/day)						
All Participants	1600 (321)	1597 (277)	1579 (280)	1604 (273)	1534 (302)	>0.05
Females	1442 (273)	1469 (218)	1446 (229)	1478 (178)	1386 (159)	>0.05
Males	1786 (271)	1768 (260)	1756 (247)	1772 (293)	1732 (338)	>0.05
REE (kcal/day/kg)						
All Participants	17.8 (3.1)	18.6 (3.7)	18.6 (3.5)	19.2 (3.8)	18.4 (3.9)	>0.05
Females	17.5 (3.0)	18.4 (4.0)	18.4 (3.8)	18.9 (3.4)	18.0 (3.9)	>0.05
Males	18.2 (3.5)	18.9 (3.3)	19.0 (3.2)	19.5 (4.5)	19.1 (4.1)	>0.05
REE (kcal/day/AFM)						
All Participants	* 53.8 (23.1)	57.4 (27.1)	60.0 (30.2)	63.5 (34.3)	* 61.8 (31.3)	<0.0001
Females	* 47.2 (23.4)	51.3 (29.4)	53.1 (31.7)	55.2 (33.2)	* 52.7 (31.2)	<0.01
Males	* 62.5 (20.3)	65.5 (22.1)	69.2 (26.3)	74.7 (33.5)	* 74.0 (27.9)	<0.01
Systolic BP (mmHg)						
All Participants	* 133 (20)	125 (16.1)	120 (15.4)	119 (16.1)	* 120 (16.8)	<0.001
Females	* 134 (16.5)	127 (16.1)	117 (14.0)	119 (12.6)	* 122 (14.0)	<0.001
Males	131 (24.8)	121 (17.8)	122 (17.3)	118 (20.6)	117 (20.2)	<0.05
Diastolic BP (mmHg)						
All Participants	* 76.3 (9.7)	72.6 (8.8)	69.0 (8.9)	70.8 (8.1)	* 70.5 (10.9)	<0.001
Females	73.7 (6.6)	70.5 (7.9)	67.3 (8.2)	68.9 (7.9)	70.1 (8.8)	<0.05
Males	* 80 (12.2)	75.7 (9.4)	71.3 (9.6)	73.4 (7.9)	* 71.1 (13.6)	<0.001

Note: Data are expressed as mean (sd) for general linear model repeated-measures ANOVA with Bonferroni adjustment for multiple comparisons. ^+^ *p* value for the effect of diet across time. * *p* < 0.05 for the difference between baseline and study end.

## Abbreviations

AFM	Absolute Fat Mass
BMI	Body Mass Index
BP	Blood Pressure
DBP	Diastolic Blood Pressure
FFM	Fat Free Mass
LBM	Lean Body Mass
REE	Resting Energy Expenditure
SBP	Systolic Blood Pressure
SMM	Skeletal Muscle Mass
TBW	Total Body Water
WHR	Waist-to-Hip Ratio
